# P001: Comparison of central line-associated bloodstream infection rates when changing to a zero fluid displacement intravenous needleless connector in acute care

**DOI:** 10.1186/2047-2994-2-S1-P1

**Published:** 2013-06-20

**Authors:** C Chernecky, D Macklin, WR Jarvis, T Joshua

**Affiliations:** 1 Georgia Regents University, Augusta, USA; 2 Consultant Vascular Access, Marietta, USA; 3 Jason & Jarvis Associates, LLC, Hilton Head, USA

## Introduction

The ability to decrease CLA-BSI has seen some improvement but more is necessary to prevent negative patient outcomes. One area that has not been researched is the actual technology or connector product and its effect on bloodstream infections.

## Objectives

Using the Healthcare and Technology Synergy (HATS) model^1^the purpose of this multicenter study was to compare central line-associated bloodstream infection (CLA-BSI) rates associated with the use of intravenous (IV) positive or negative (including mechanical valve and split septum devices) pressure needleless connectors to a zero fluid displacement needleless connector.

## Methods

Quasi-experimental study over 140 months, 5 states, six specialty settings (3 intensive care units (ICU), and one each of medical ICU, surgical ICU, and Long Term Acute Care -LTAC) comparing CLA-BSI rates associated with positive (6,649 catheter-days) or negative (17,810 catheter-days) IV needleless connectors. There were a total of 24,459 pre-zero fluid displacement catheter-days over 70 months compared to 25,621 total post zero displacement connector catheter-days over 70 months. Paired t-tests were used to examine differences between catheter-days and CLA-BSI rates before and after zero fluid displacement connector adoption. Statistical significance was assessed using an alpha level of 0.05.

## Results

The number of catheter days was similar both before and after zero fluid displacement connector adoption. There was a statistically significant higher CLA-BSI rate when either negative (p = 0.039) or positive (p = 0.0158) pressure mechanical IV connectors were used. Overall, a decrease in CLA-BSIs per 1000 catheter days was found after changing from negative, or positive IV connectors to the zero fluid displacement connector (p = 0.005).

## Conclusion

We documented a statistically significant decrease in CLA-BSI rates when either a negative or positive IV needleless connectors were changed to a zero fluid displacement connector in multiple acute settings. The data reveals that IV needleless connector design impacts CLA-BSI rates and product is a significant variable in the HATS model for comparative effectiveness.

## Disclosure of interest

C. Chernecky: None declared, D. Macklin Shareholder of RyMed Technologies Inc, Consultant for RyMed Technologies Inc, W. Jarvis: None declared, T. Joshua: None declared

